# Morphological divergence between three Arctic charr morphs – the significance of the deep-water environment

**DOI:** 10.1002/ece3.1573

**Published:** 2015-07-14

**Authors:** Sigrid Skoglund, Anna Siwertsson, Per-Arne Amundsen, Rune Knudsen

**Affiliations:** Department of Arctic and Marine Biology, University of TromsøN-9037, Tromsø, Norway

**Keywords:** Geometric morphometrics, incipient speciation, phenotypic diversity, profundal piscivore, resource polymorphism, salmonid

## Abstract

Morphological divergence was evident among three sympatric morphs of Arctic charr (*Salvelinus alpinus* (L.)) that are ecologically diverged along the shallow-, deep-water resource axis in a subarctic postglacial lake (Norway). The two deep-water (profundal) spawning morphs, a benthivore (PB-morph) and a piscivore (PP-morph), have evolved under identical abiotic conditions with constant low light and temperature levels in their deep-water habitat, and were morphologically most similar. However, they differed in important head traits (e.g., eye and mouth size) related to their different diet specializations. The small-sized PB-morph had a paedomorphic appearance with a blunt head shape, large eyes, and a deep body shape adapted to their profundal lifestyle feeding on submerged benthos from soft, deep-water sediments. The PP-morph had a robust head, large mouth with numerous teeth, and an elongated body shape strongly related to their piscivorous behavior. The littoral spawning omnivore morph (LO-morph) predominantly utilizes the shallow benthic–pelagic habitat and food resources. Compared to the deep-water morphs, the LO-morph had smaller head relative to body size. The LO-morph exhibited traits typical for both shallow-water benthic feeding (e.g., large body depths and small eyes) and planktivorous feeding in the pelagic habitat (e.g., streamlined body shape and small mouth). The development of morphological differences within the same deep-water habitat for the PB- and PP-morphs highlights the potential of biotic factors and ecological interactions to promote further divergence in the evolution of polymorphism in a tentative incipient speciation process. The diversity of deep-water charr in this study represents a novelty in the Arctic charr polymorphism as a truly deep-water piscivore morph has to our knowledge not been described elsewhere.

## Introduction

Polymorphic populations are found in many freshwater fish taxa such as salmonids, cichlids, and sticklebacks (Robinson and Parsons 2002). Resource polymorphism typically occurs in phenotypically plastic species, where the individuals have the ability to change their phenotype in response to environmental changes (Skúlason and Smith 1995; West-Eberhard 1989). The phenomenon is characterized by the occurrence of distinct morphs showing differential niche use, usually through discrete differences in feeding ecology and habitat use. A persistent divergent selection related to different ecological factors may promote different alternative phenotypes that may form the basis for an ecologically driven speciation process (Schluter 2001, 2009; Rundle and Nosil 2005; Sobel et al. 2010). The ecological factors may include differences in the environment and/or ecological interactions related to resource acquisition (e.g., habitat preference and/or prey selection) (Schluter 2001).

Important aspects of the ecological niche use and behavior of fishes are reflected in their morphology, as form and function are highly related (Webb 1984; Wootton 1998). In general, the body shape of fish is closely related to habitat complexity and swimming behavior (Schoener 1971; Webb 1984), and the head shape to foraging and prey specializations (Snorrason et al. 1994; Adams et al. 1998). Typically, planktivore fish have a pointed head shape with a terminal positioned mouth and a streamlined body shape adapted for pursuing and capturing zooplankton prey in the pelagic habitat (Webb 1984; Jonsson and Jonsson 2001; Robinson and Parsons 2002; Harrod et al. 2010). Benthivore fish usually have a more rounded head shape with a small subterminally positioned mouth and a short and deep laterally compressed body shape with long pectoral fins, adapted to capture invertebrates in a more complex benthic habitat (Jonsson and Jonsson 2001; Harrod et al. 2010; Knudsen et al. 2011). Piscivore fish, commonly have an elongated body shape and a large, pointy head with a big terminal mouth well suited to capturing smaller fish in the water column (Skúlason et al. 1989; Adams et al. 1998; Jonsson and Jonsson 2001).

This study describes the morphological diversity of three recently identified sympatric fish morphs of a highly plastic postglacial fish species, Arctic charr *Salvelinus alpinus* (L.). Furthermore, we discuss how different morphological specializations may relate to differences in physical characteristics and different resource utilization within the contrasting deep-water and upper-layer environments. Arctic charr is a well-documented polymorphic fish species with high phenotypic plasticity, existing both in resident (nonmigratory) and anadromous populations (Jonsson and Jonsson 2001; Klemetsen 2010). Polymorphic lacustrine populations of Arctic charr typically display two to four sympatric morphs that differ in habitat utilization and diet, morphology, and life-history characteristics (e.g., growth pattern, relative reproductive effort, and age and size at maturity) (Sandlund et al. 1992; Adams et al. 2003; Klemetsen 2010). The most common pattern of divergence in polymorphic Arctic charr populations is along the benthic–limnetic resource axis (Jonsson and Jonsson 2001), including classic examples from Thingavallavatn, Iceland (Malmquist et al. 1992; Sandlund et al. 1992) and Loch Rannoch, Scotland (Adams et al. 1998). A few Arctic charr studies have found diversification along the depth gradient of lakes (Klemetsen 2010), as, for example, in the subarctic lake Fjellfrøsvatn, Norway, where two genetically and morphologically distinct morphs specialize on shallow-water (littoral and pelagic) and deep-water (profundal) resources (Klemetsen et al. 1997, 2002; Westgaard et al. 2004; Knudsen et al. 2006; Amundsen et al. 2008). Deep (depth > 20 m) postglacial lakes show sharp contrasts along the depth axis. The littoral and pelagic environments have higher light regimes and summer temperatures that vary daily and seasonally. Furthermore, they offer diverse and rich food resources (littoral benthos and zooplankton) and harbor several predators (fish and birds). In contrast, the profundal environment is monotonous with low physical complexity dominated by fine soft bottom sediments and no vegetation. Temperatures are uniform, and light is low or absent. This habitat is often used as a predatory refuge by juvenile fish (Klemetsen et al. 1989).

Recently, two Arctic charr morphs adapted to the deep-water habitat were identified in a Norwegian subarctic lake (Skogsfjordvatn), coexisting with a third morph mainly residing in the more commonly utilized shallow-water habitats (Smalås et al. 2013). The three morphs are named from their observed spawning habitat and main prey resource use. The littoral spawning omnivore morph (hereafter referred to as the LO-morph) predominantly utilizes the shallow-water layer resources in the littoral and pelagic zones. Immature individuals of the LO-morph have a silvery color with light spots on the lateral sides and a darker dorsal side, while mature individuals have typical spawning coloration with a red-orange belly and white edges on the paired fins. The two profundal spawning morphs utilize different prey items, including a benthivore morph (the PB-morph) and a piscivore morph (the PP-morph), and have highly contrasting life-history traits (growth rates, adult sizes, and age and size at maturity) (Smalås et al. 2013). The profundal slow-growing PB-morphs have the appearance of a young charr with a pale yellow coloration and pale brown parr marks. They reach maturity at a young age (∼3 years) and small body size (∼8.5 cm) and have no specific spawning coloration. The PP-morphs have an elongated body with a generally large head. They are less colorful compared to the similar sized LO-morph, ranging from relatively pale to completely dark with no clear red spawning colors. The PP-morphs are relatively slow-growing, but reach the largest body size of all morphs (40.3 cm). They mature at an old age (∼9.2 years) and large body size (∼26 cm). To our knowledge, this represents the only documentation of two co-occurring deep-water Arctic charr morphs.

In this study, we discuss whether resource (i.e., habitat and diet)-driven adaptations are present in the morphology of the three sympatric charr morphs. Based on the differences in life-history traits and ecology, we expected all three morphs in Skogsfjordvatn to be morphologically different. The two profundal specialist morphs were expected to be morphologically more similar to each other than to the LO-morph, as they have evolved in an identical and uniform abiotic environment (the deep-water habitat) with low light and temperature levels throughout all seasons. Because of the low light conditions in the profundal habitat, we predicted the two profundal morphs to have relative large eyes. We also expect them to differ in morphological traits related to their contrasting diet (benthivory vs. piscivory) use. As the PP-morph is consuming fish that are much larger in size than profundal invertebrates, this morph is expected to have the largest mouth compared with the other sympatric morphs. The LO-morph was expected to differ from the deep-water morphs in morphological traits related to both habitat and diet utilization.

## Materials and Methods

Skogsfjordvatn (69.95°N, 19.17°E) is a deep (maximum depth 100 m), oligotrophic dimictic lake in northern Norway, with a surface area of 13.6 km^2^. The lake is situated 20 m a.s.l. close to the ocean (1 km distance) and was originally a marine fjord (Bratrein 1989). The drainage area varies from high alpine landscapes to lowlands dominated by birch forest, marches, and heather. The present fish community consists of both anadromous and resident populations of Arctic charr and brown trout (*Salmo trutta*), anadromous Atlantic salmon (*S. salar*), three-spined stickleback (*Gasterosteus aculeatus*), and occasionally catadromous European eel (*Anguilla anguilla*).

Fish were sampled in May, June, and August 2011 in all major lake habitats (littoral, pelagic and profundal) using multipaneled gillnets with mesh sizes from 5 to 55 mm. Bottom gillnets (30 × 1.5 m) were set in the littoral zone (0–15 m) and at three different depths of the profundal zone (25, 35 and 45 m), and floating nets (40 × 6 m) were set in the pelagic zone. Gillnets were set in the afternoon and collected in the next morning. For more details of the fish sampling, see Smalås et al. (2013). During field sampling, the temperature in the littoral/pelagic zone (1 m depth) ranged from 3.5°C in May (full spring circulation) to 12.0 and 13.3°C in June and August, respectively. In the profundal zone (≥20 m depth), the temperature was 7.0°C in both June and August. The Secchi depth was measured to be 16.5 m in May and 14.5 m in June and August.

**Figure 1:**
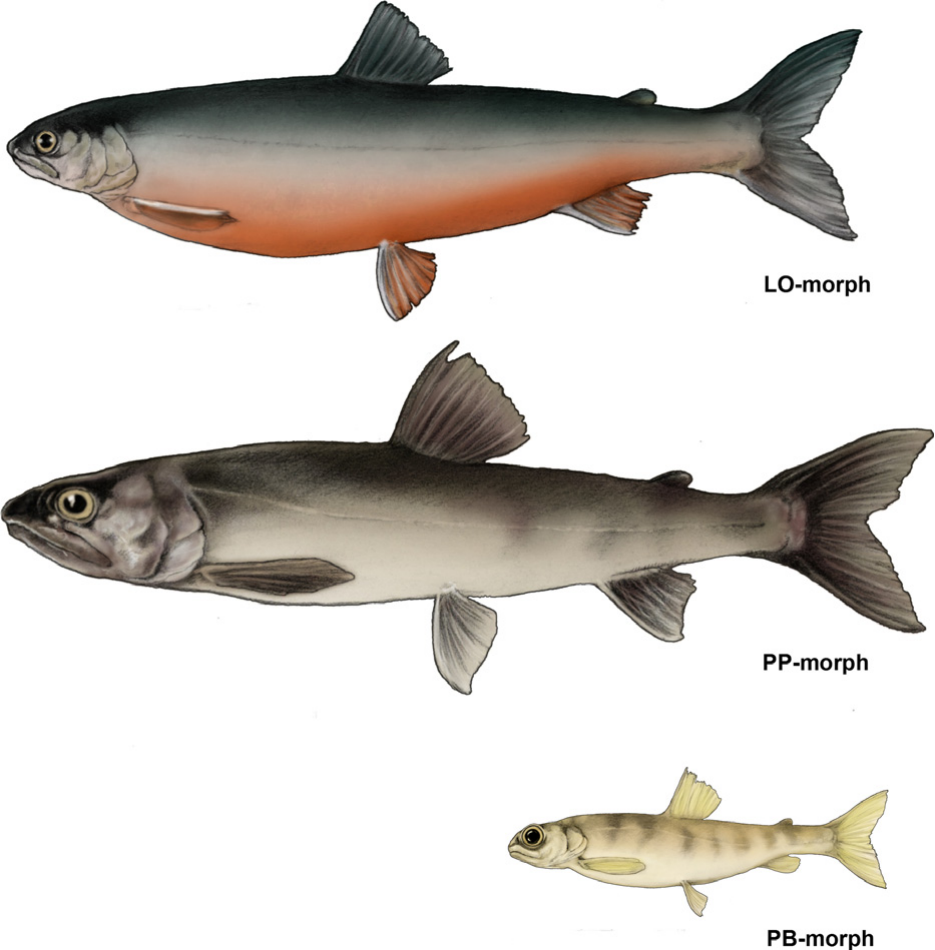
Color drawings showing the typical appearance of the three Arctic charr morphs from Skogsfjordvatnet: the littoral spawning omnivore morph (LO-morph), the profundal spawning piscivore morph (PP-morph), and the profundal spawning benthivore morph (PB-morph).

In the field, Arctic charr were subjectively sorted into the three different morphs (LO-, PB-, and PP-morph) based on their general appearance. Drawings of the three Arctic charr morphs were made according to general observations in field and while photographing the charr (Fig.1). Identification was mainly associated with differences in head and body morphology and coloration combined with sexual maturation in smaller individuals. Altogether, 61 individuals of the LO-morph (mean fork length: 24.8 cm, range: 18.7–31.9 cm), 47 of the PB-morph (mean: 10.7 cm, range: 7.8–13.7 cm), and 51 of the PP-morph (mean: 25.4 cm, range: 10.1–44.8 cm) were included in the morphological analyses (see Fig.8 for size distributions).

Prey items in the stomach contents of individual fish (*N = *159) were identified and sorted into six prey groups including the following: (1) zooplankton (limnetic cladocerans and copepods); (2) littoral benthos (*Lymnea peregra*, *Gammarus lacustris*, mayfly, caddisfly, stonefly, and chironomid larvae); (3) pleuston (mainly adult terrestrial insects); (4) chironomid pupae; (5) profundal prey (the semibenthic chydorid cladoceran *Eurycercus lamellatus*; pea mussels *Pisidium* sp., chironomid larvae, and the benthic copepod *Acanthocyclops gigas*); and (6) fish. Chironomid larvae from littoral caught fish were regarded as littoral prey, while from profundal caught fish regarded as profundal prey. The abundance of each prey group (*A*_*i*_) was estimated by the formula: % *A*_*i*_* = *∑(S_*i*_/S_*t*_) × 100, where S_*i*_ is the stomach content composed by prey *i,* and S_*t*_ is the total stomach content in the sample (Amundsen et al. 1996).

**Figure 2:**
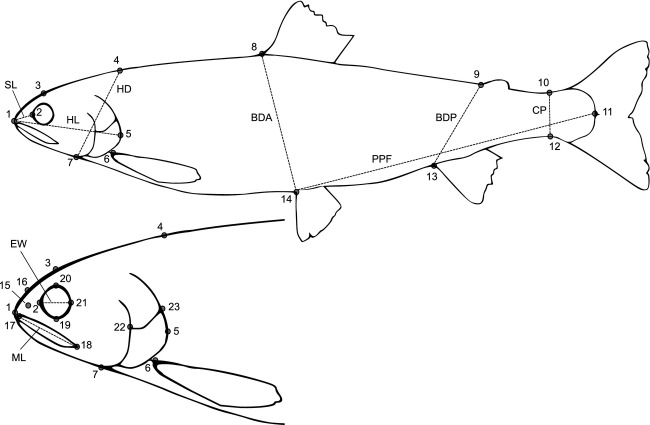
Landmark positions used in geometric morphometrics and measurements of linear morphological traits. Landmarks used for analyses of body shape: 1: Anterior point of the snout, 2: anterior extreme of bony orbit of the eye, 3: top of cranium at midpoint of eye, 4: top of cranium at posterior point of the bony opercle (5), 5: posterior point of the bony opercle, 6: dorsal insertion of pectoral fin, 7: anterioventral point of bony opercle, 8: anterior insertion of dorsal fin, 9: anterior base of adipose fin, 10: dorsal origin of caudal fin membrane, 11: posterior border of the hypural bones at the lateral midline, 12: ventral origin of caudal fin membrane, 13: anterior insertion of anal fin, and 14: anterior insertion of pelvic fin. Landmarks used for head shape: 1–7, and 15: Center of nostril, 16: top of cranium at midpoint of nostril (15), 17: anterior point of the upper jaw, 18: posterior point of the upper jaw, 19: ventral extreme of bony orbit of the eye, 20: dorsal extreme of bony orbit of the eye, 21: posterior extreme of bony orbit of the eye, 22: ventral point of intersection between the opercle and preopercle bones, 23: posterior point of intersection between the opercle and subopercle bones. Interlandmark distances used for linear morphological traits: caudal peduncle depth (CP): 10–12, body depth posterior (BSP): 9–13, body depth anterior (BDA): 8–14, postpelvic fin length (PPF): 11–14, head depth (HD): 4–7, head length (HL): 1–5, snout length (SL): 1–2, eye width (EW): 2–21, maxilla length (ML): 17–18.

The left side of each fish was photographed with a digital camera (Nikon Coolpix 5400) (see Siwertsson et al. 2013 for further details). Landmarks were digitized on 23 anatomical locations using tpsDig v.2.16 (Rohlf 2010) and were used for both landmark-based geometric morphometrics (Rohlf and Marcus 1993; Bookstein 1997; Adams et al. 2004; Slice 2007) and traditional morphometric analysis (linear measurements) (Fig.2).

### Geometric morphometrics

We performed separate geometric morphometric analyses of the head (16 landmarks) and body shape (14 landmarks) of the fish. To standardize the landmark coordinates and remove the nonshape effects of size, position, and orientation of each specimen, a general Procrustes analysis (GPA) was performed in MorphoJ v.1.06b (Klingenberg 2011). A GPA results in a new set of landmark coordinates, Procrustes Coordinatesc used to describe the shape variation (Bookstein 1997; Adams et al. 2004; Slice 2007). To explore the morphological variation between individuals of Arctic charr in Skogsfjordvatn, we performed principal component analyses (PCA) of shape variables (Procrustes Coordinates) in MorphoJ. To graphically illustrate variation in body and head shape along the resulting principal component (PC) axes, wireframe outlines of extreme shapes along each axis were created in MorphoJ.

To test for morphological differences between the three morphs, we performed multivariate analyses of variances (MANOVAs) using individual scores on the first five PC axes in each of the PCAs as dependent variables. The approximate F-values from pairwise MANOVAs were used to indicate the magnitude of shape differences between the morphs. Differences between morphs were also explored for each of these PC axes separately using analysis of variances (ANOVAs) to get more detailed knowledge about the morphological variation. ANOVAs with significant morph effects were followed up by post hoc Tukey’s HSD tests to identify which of the morphs were significantly different from each other. MANOVA, ANOVA, and post hoc tests were performed in the program R (R Development Core Team 2012).

### Linear measurements

Nine linear morphological traits were measured as the distance between specific landmark pairs on each fish (Fig.1). These traits were selected based on previous studies of littoral and profundal morph pairs of Arctic charr, European whitefish (*Coregonus lavaretus),* and lake charr (*Salvelinus namaycush*) (Klemetsen et al. 2002; Kahilainen and Østbye 2006; Zimmerman et al. 2006; Siwertsson et al. 2013). Three of the traits (snout length, maxilla length and eye diameter) have been shown to have a genetic basis in littoral and profundal spawning Arctic charr morphs from Fjellfrøsvatn (Klemetsen et al. 2002). Calculations of distances between landmarks were made using an internet-accessible landmark measurement tool (Krieger 2006). All measurements were allometrically aligned to the grand mean fork length 19.5 cm. First, all morphological trait values were log_10_-transformed to reduce heterogeneity in variance. Second, the traits were size-adjusted using the allometric growth formula (Senar et al. 1994):

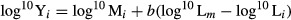
where Y_*i*_ is the size-adjusted trait value, M_*i*_ is the measured trait value, *b* is the linear regression coefficient (slope) of the measured trait (log^10^M_*i*_) against fork length (log^10^ L_*i*_) within each morph, L_*i*_ is the measured fork length, and L_*m*_ is the average fork length of all fish.

Morphological differences between morphs were explored using ANOVAs (with post hoc Tukey’s HSD) for each individual trait to get a more detailed knowledge about the morphological differences between the morphs.

## Results

### Diet

**Figure 3:**
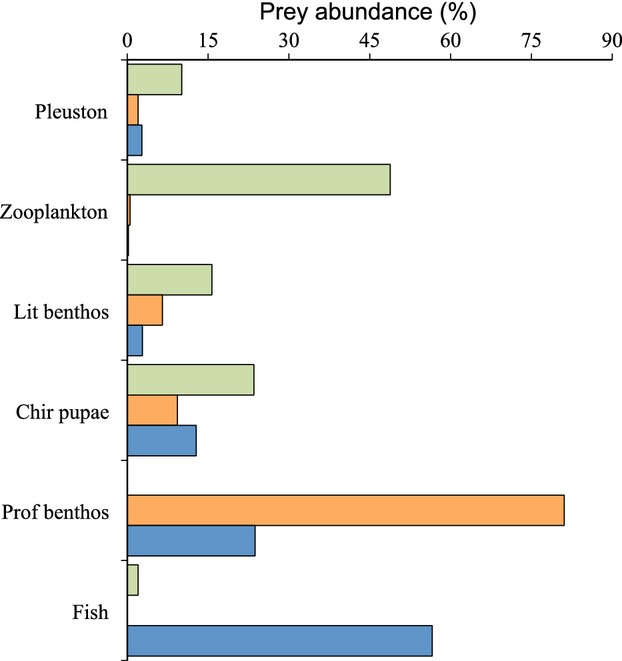
Diet (prey abundance, %) of the three Arctic charr morphs; the LO-morph (green bars), the PB-morph (orange bars), and the PP-morph (blue bars) from Skogsfjordvatn.

The diet was different between the three morphs. The LO-morph had a dominance of upper-water prey items such as zooplankton, littoral benthos, pleuston, and chironomid pupae (Fig.3). In contrast, the PB-morph had predominantly fed on profundal benthos, while the PP-morph had a dominance of fish as prey (Fig.3). Furthermore, smaller individuals of the PP-morph had also eaten profundal benthos.

### Geometric morphometrics

**Figure 4:**
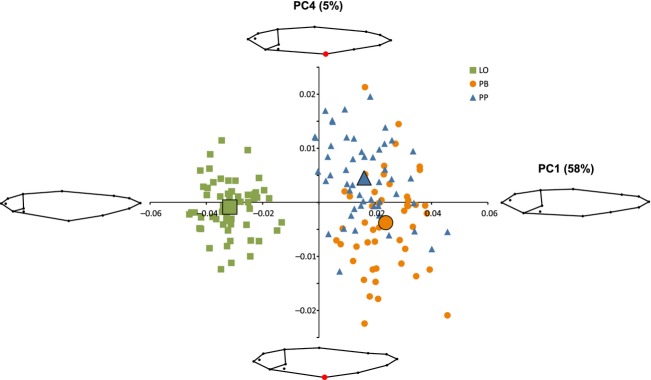
Principal component analysis of body shape (PC1 and PC4) in three morphs of Arctic charr. Mean values for each morph are illustrated by the larger symbols. Graphical illustrations show body shape at each extreme value on both axes (PC1: 0.05 and −0.05, PC4: 0.025 and −0.025). The red dot illustrates the position of the pelvic fin on extreme values of PC4.

To explore the differences in body shape between morphs in more detail, we studied the first five PC axes separately. The first PC axis (58% of total variation) was mainly based on contrasts in head size and revealed clear and significant differences between all three morphs (Table1, Fig.4). The LO-morph had a smaller head relative to body length. Although significant, the difference between the two profundal morphs (PP and PB) was very small (Table1, Fig.4). The second PC axis (12% of total variation) was mainly associated with the bending of the fish; an unwanted effect when photographing fish and this is therefore not considered any further (Fig.9). The third PC axis (7% of total variation) was mainly associated with changes in body depth (large individual variation) and did not significantly separate the different morphs (Table1, Fig.9). The piscivorous morph (PP) had significantly higher values on the fourth PC axis (5% of total variation), related to a more posterior position of the pelvic fin (Table1, Fig.4). The fifth PC axis (4% of total variation) was mainly associated with head morphology and significantly separated all three morphs (Table1, Fig.8). The PB-morph had a more steep curvature from the head to the snout, and consequently, the eye was positioned closer to the anterior part of the head. The PP-morph had the longest snout and also the longest distance from the snout to the eye.

**Table 1:** Explained variance (% of total variance) of PC1–PC5 from PCAs of body shape and head shape. Also shown are results from ANOVAs of scores on the first five PC axes. The differences in mean values between the morph pairs were explored using Tukey’s HSD tests. Statistical significance is indicated by stars: ^*^^*^^*^P < 0.001, ^*^^*^P < 0.01, ^*^P < 0.05

	PC	Explained	ANOVA		Difference between morphs		
	Axis	Variance (%)	*F*_2,156_	*P*-value	PB-LO	PP-LO	PP-PB
Body shape	PC1	57.7	586.5	<0.001	0.055^*^^*^^*^	0.048^*^^*^^*^	−0.008^*^^*^^*^
	PC2	11.7	4.6	0.012	−0.005	0.002	0.007^*^^*^
	PC3	6.9	0.8	0.46	0.001	0.002	0.001
	PC4	4.8	17.5	<0.001	−0.003	0.005^*^^*^^*^	0.008^*^^*^^*^
	PC5	3.7	18.7	<0.001	0.004^*^^*^	−0.003^*^	−0.008^*^^*^^*^
Head shape	PC1	36.0	334.0	<0.001	−0.098^*^^*^^*^	−0.053^*^^*^^*^	0.045^*^^*^^*^
	PC2	16.2	115.3	<0.001	−0.000	0.050^*^^*^^*^	0.050^*^^*^^*^
	PC3	10.0	0.6	0.57	−0.003	−0.005	−0.002
	PC4	7.4	1.4	0.26	0.002	−0.004	−0.007
	PC5	5.1	6.0	0.003	0.004	−0.008^*^	−0.011^*^^*^

MANOVA of individual scores on PC1–PC5 showed that there was an overall difference in body shape among the three morphs (MANOVA: approx. *F*_2,156_* = *60.0, *P* < 0.001). The difference was largest between the PB- and LO-morph (MANOVA: approx. *F*_1,106_* = *310.1, *P* < 0.001), while the two profundal morphs (PB and PP) were the most similar (MANOVA: approx. *F*_1,96_* = *22.4, *P* < 0.001). The body shape difference between the PP- and LO-morph was intermediate (MANOVA: approx. *F*_1,110_* = *183.4, *P* < 0.001).

**Figure 5:**
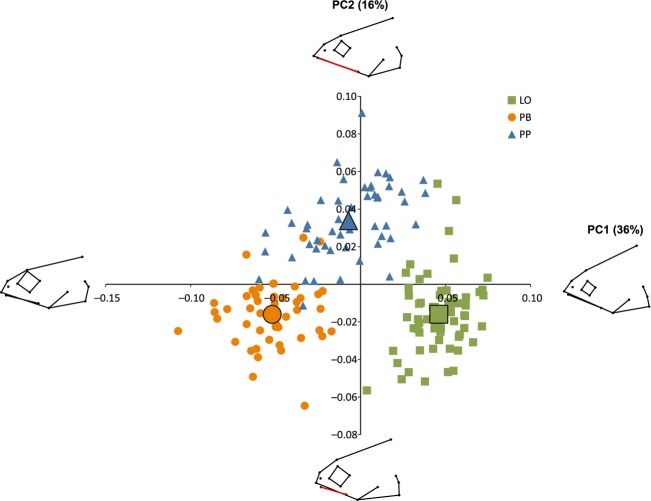
Principal component analysis of head shape (PC1 and PC2) in three morphs of Arctic charr. Mean values for each morph are illustrated by the larger symbols. Graphical illustrations show head shape at each extreme value on both axes (PC1: 0.09 and −0.09, PC4: 0.07 and −0.07). The red line indicates the size of the upper maxilla on extreme values of PC2.

In the PCA of head shape, the first PC axis accounted for 36% of the total morphological variation. PC 1 mainly represents opposite contrasts in head depth and eye size and significantly separated all three morphs (Table1, Fig.5). The LO-morph had relatively large head depth and a small eye size, while the PB-morph had a more narrow head shape and larger eyes. The second PC axis (16% of total variation) mainly represented contrasts in snout length and maxilla length. The PP-morph differed significantly from the PB- and LO-morph (Table1, Fig.5), as it had a more pointed head shape, with longer snout and a longer maxillary bone, and also a slightly narrower head shape and smaller eyes. The PP-morph was different from the two other morphs also on the fifth PC axis (5% of total variation), associated with similar morphological characteristics as the second PC axis, for example, more narrow and pointy head shape with longer snout and smaller eyes (Table1, Fig.10). The third and fourth PC axes explained 10% and 7% of the total variation, but there were no significant differences among the morphs (Table1, Fig.10).

MANOVA of individual scores on the first five PC axes showed that the three morphs also had significantly different head shape (MANOVA: approx. *F*_2,156_* = *48.3, *P* < 0.001). The PB- and LO-morph were the most different (MANOVA: approx. *F*_2,106_* = *236.9, *P* < 0.001), while the two profundal morphs had the most similar head shapes (MANOVA: approx. *F*_2,96_* = *48.5, *P* < 0.001). The PP- and LO-morph had intermediate head shape difference (MANOVA: approx. *F*_2,110_* = *134.7, *P* < 0.001).

### Linear measurements

**Figure 6:**
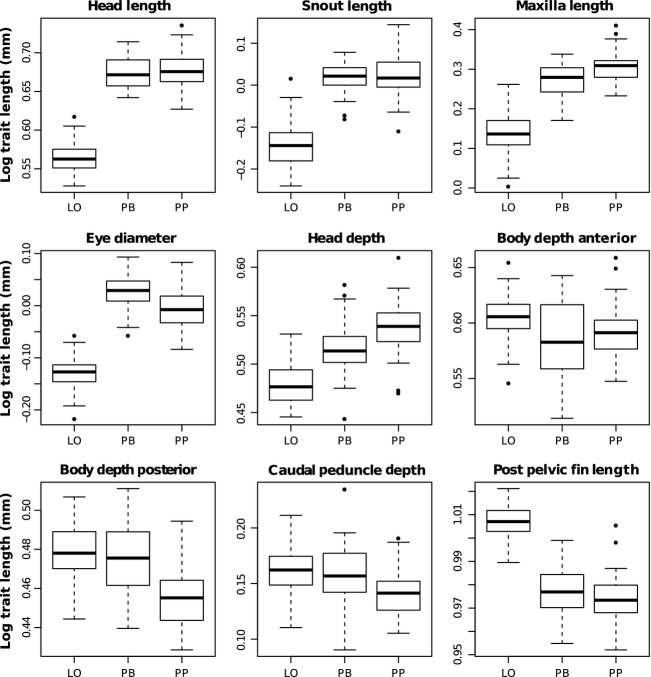
Box plots of size-corrected linear traits for the three Arctic charr morphs; the littoral spawning omnivore morph (LO-morph), the profundal spawning piscivore morph (PP-morph), and the profundal spawning benthivore morph (PB-morph).

All nine linear morphological traits were significantly different between morphs (ANOVA, *P* < 0.001, Table2). The PP- and LO-morph were the most different, with all nine traits showing significant differences. The PP-morph had a larger head and slimmer body than the LO-morph. More specifically, the PP-morph had larger head traits (head length, depth, maxilla length, snout length, and eye size), smaller body depths and shorter distance from the pelvic fin to the caudal fin base (PPF) compared to the LO-morph (Fig.6). The PB-morph also had significantly larger head traits compared to the LO-morph, but their body shapes seemed to be more similar. Only the anterior body depth (BDA) and the posterior pelvic fin (PPF) distances were significantly shorter in the PB-morph compared to the LO-morph. Although the PP-morph and the PB-morph showed similar morphological differences compared to the LO-morph, they were also different. The PP-morph had significantly larger head depth and maxilla length, and smaller eyes. The PP-morph was also slimmer in the posterior body shape (BDP and CP).

**Table 2:** Results from ANOVAs of nine size-adjusted traits and post hoc Tukey’s HSD tests indicated significant differences between morphs. The observed direction of trait differences is indicated for each trait. Significant differences of trait means between two morphs are indicated by stars: ^*^^*^^*^P < 0.001, ^*^^*^P > 0.01, ^*^P > 0.05

Measured morphological trait		ANOVA		Difference between morphs			Observed direction of trait difference
		*F*_2,156_	*P*-value	PP-LO	PB-LO	PP-PB	
Head length	HL	479.3	<0.001	0.110^*^^*^^*^	0.109^*^^*^^*^	0.001	PP = PB > LO
Snout length	SL	223.2	<0.001	0.161^*^^*^^*^	0.157^*^^*^^*^	0.004	PP = PB > LO
Maxilla length	ML	224.3	<0.001	0.166^*^^*^^*^	0.134^*^^*^^*^	0.032^*^^*^	PP > PB > LO
Eye diameter	ED	368.7	<0.001	0.122^*^^*^^*^	0.155^*^^*^^*^	−0.033^*^^*^^*^	PB > PP > LO
Head depth	HD	86.3	<0.001	0.058^*^^*^^*^	0.036^*^^*^^*^	0.022^*^^*^^*^	PP > PB > LO
Body depth anterior	BDA	9.3	<0.001	−0.014^*^^*^	−0.020^*^^*^^*^	0.006	LO > PP = PB
Body depth posterior	BDP	29.0	<0.001	−0.023^*^^*^^*^	−0.003	−0.020^*^^*^^*^	LO = PB > PP
Caudal peduncle depth	CPD	13.6	<0.001	−0.022^*^^*^^*^	−0.005	−0.017^*^^*^^*^	LO = PB > PP
Postpelvic fin length	PPF	216.7	<0.001	−0.033^*^^*^^*^	−0.029^*^^*^^*^	−0.004	LO > PB = PP

**Figure 7:**
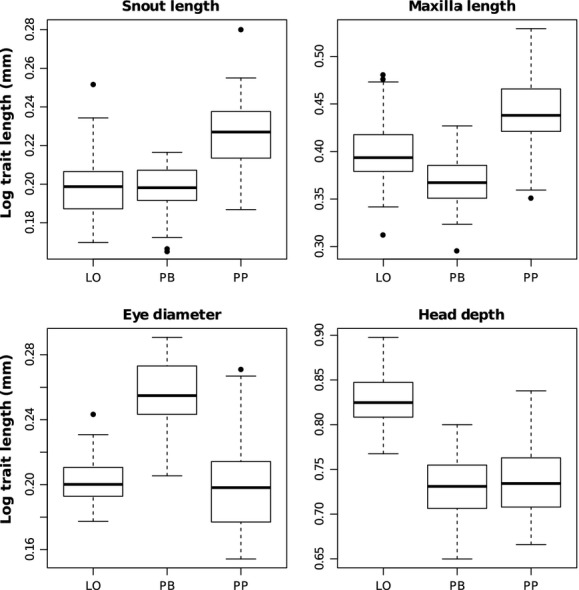
Box plots illustrating differences between morphs (LO, PB, and PP) in four head traits accounting for differences in head size (length).

**Figure 8:**
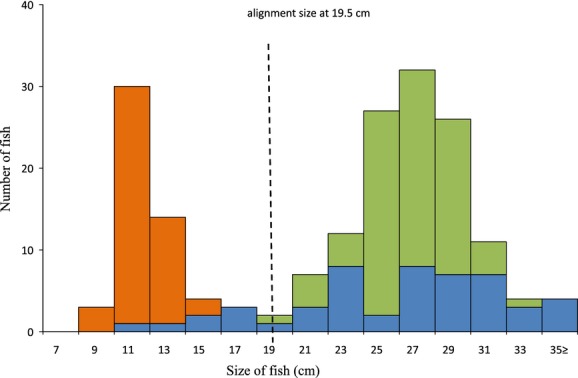
Length (in cm) distribution of the three different morphs (LO-morph in green, PB-morph in orange and PP-morph in blue) in Skogsfjordvatn with alignment size at 19.5 cm given.

**Figure 9:**
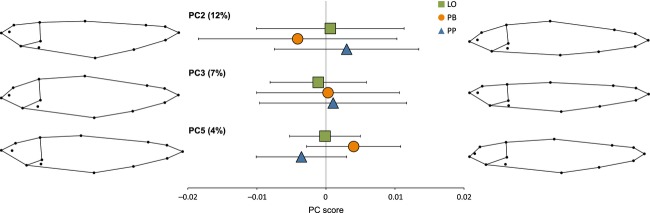
Mean and standard deviation for the three morphs of Arctic charr for PC2, 3, and 5 from a principal component analysis of body shape. Illustrations show the body shape associated with individual extreme values on PC2 (0.03 and −0.03), PC3 (0.03 and −0.03), and PC5 (0.025 and −0.025).

**Figure 10:**
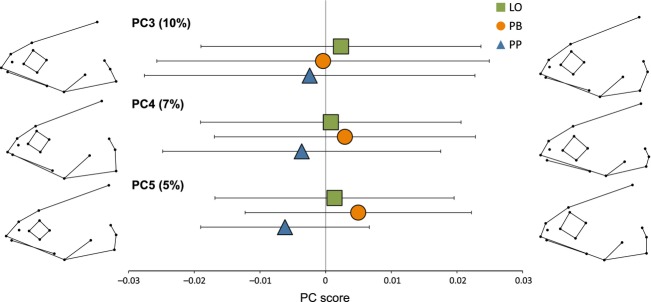
Mean and standard deviation for the three morphs of Arctic charr for PC3, PC4, and PC5 from a principal component analysis of head shape. Illustrations show the head shape associated with individual extreme values at 0.06 and −0.06 for all three PC axes.

Several of the head measurements seemed to follow a general correlation with head length, with the two profundal morphs always having larger heads and head traits than the LO-morph. To account for differences in head size when comparing these traits between morphs, we used the ratio of each trait to the individual head length (Fig.7, Table3). The PP-morph was characterized by a slim head with a large mouth and eyes positioned more posterior than the other morphs. The PB-morph also had a slim head, but a small mouth and large eyes positioned closer to the snout. The LO-morph had the largest head depth, medium sized mouth, and eyes positioned closer to the snout.

**Table 3:** Results from ANOVA and post hoc Tukey’s HSD tests indicated significant differences between morphs for four head traits when accounting for head length (ratios). Significant differences of ratio means between two morphs are indicated by stars: ^*^^*^^*^P < 0.001, ^*^^*^P > 0.01, ^*^P > 0.05

Measured morphological trait		ANOVA		Difference between morphs		
		*F*_2,156_	*P*-value	PP-LO	PB-LO	PP-PB
Snout length	SL	48.1	<0.001	0.027^*^^*^^*^	0.000	0.027^*^^*^^*^
Maxilla length	ML	63.9	<0.001	0.042^*^^*^^*^	−0.030^*^^*^^*^	0.072^*^^*^^*^
Eye diameter	ED	104.8	<0.001	−0.003	0.053^*^^*^^*^	−0.056^*^^*^^*^
Head depth	HD	140.8	<0.001	−0.092^*^^*^^*^	−0.100^*^^*^^*^	0.008

## Discussion

The present study revealed clear significant morphological differences among the three Arctic charr morphs in Skogsfjordvatn. The two profundal morphs (PP and PB) were generally more similar to each other than to the littoral morph (LO), despite their large differences in size (Smalås et al. 2013, this study). These two deep-water morphs have evolved under identical abiotic conditions with constant low light and temperature and a uniform physical habitat and have an overall similarity in morphological adaptations in accordance with our expectation. Compared to the shallow-water LO-morph, the profundal morphs had larger heads compared to body size, slimmer head shapes, and shorter posterior pelvic fin length. Although general head shape was most similar between the two profundal morphs, there were also some specific differences in their head characteristics (e.g., eye and mouth size), which may reflect their different diet specializations. Hence, the highly diverged but narrower diet niches of the two profundal spawning morphs compared to the LO-morph have apparently resulted in specialized trophic morphological traits.

The profundal piscivorous PP-morph was characterized by an elongated head shape with a pointed, long snout, and a large mouth. This is in sharp contrast to the blunt head shape typically observed in small-sized profundal charr (e.g., Klemetsen 2010; this study). The long, robust head and the large mouth with numerous big teeth of the PP-morph furthermore imply highly specialized adaptations to fish predation (Nilsson and Brönmark 2000); characteristics that also have been observed in other piscivorous Arctic charr morphs (Sandlund et al. 1992; Snorrason et al. 1994; Adams et al. 1998; Fraser et al. 1998; Power et al. 2005, 2009). In addition, compared to the shallow-water LO-morph, the PP-morph had significantly larger eyes at similar body sizes. Large eye size has also been observed in other shallow-water piscivorous morphs of Arctic charr (Sandlund et al. 1992; Adams et al. 1998) and is likely related to their predacious behavior and the ability to locate small evasive fish at larger distances (e.g., Gartner et al. 1997). The PP-morph also had an elongated body shape (slimmer posterior body and caudal peduncle depths) and more posterior position of the pelvic fin. These are traits that may help to reduce drag, increase acceleration, and improve thrust motion, which constitute good adaptations to capture evasive prey such as small fish (Webb 1984; Svanbäck 2004). In Arctic charr, piscivore behavior is often a result of ontogenetic transformations commonly found in allopatric populations and in a few polymorphic populations (Amundsen 1994; Snorrason et al. 1994; Adams et al. 1998; Mittelbach and Persson 1998). There are at least two different ontogenetic pathways to piscivory in Arctic charr, leading to planktivore-like (Skúlason et al. 1989; Arbour et al. 2011) or benthivore-like piscivorous morphologies (Fraser et al. 1998). The similarity of the PP-morph in Skogfjordvatn to the profundal benthivorous (PB) morph indicates a bentivore-like morphology, but it also has some traits associated to the pelagic habitat such as elongated head and body shape (Webb 1984; Jonsson and Jonsson 2001; Robinson and Parsons 2002). Piscivore charr morphs are usually located in the upper-water layer preying on smaller fish in the pelagic or littoral habitats (Sandlund et al. 1992; Adams et al. 1998; Power et al. 2005, 2009). In contrast, the piscivore morph in Skogsfjordvatn apparently resides in the profundal habitat throughout its lifetime and was only infrequently caught at shallow water above 20 meters depth (Smalås et al. 2013). Development of piscivore behavior in Arctic charr normally occurs in lakes with suitable density of prey species and low interspecific competition from other piscivore fish species (Jonsson and Jonsson 2001). In Skogsfjordvatn, the piscivore niche in the upper-water layer is predominantly occupied by brown trout as for other lakes in this region (Persson et al. 2007; Eloranta et al. 2013), while Arctic charr is the only fish species caught in the profundal zone. With this in mind, it is reasonable to assume that the emergence of a small-sized profundal benthivore morph residing in the deep-water habitat at all seasons and representing a stable resource in the lean profundal environment, has contributed to the ecological opportunity for a profundal piscivorous morph to evolve.

The profundal benthivorous PB-morph was mainly characterized by a relatively large head, with large eyes and steep curvature of the snout (rounded head shape). Compared to the PP-morph, the body shape was deeper and small sized. These characteristics imply adaptations to a benthic lifestyle including utilization of small-sized benthic prey submerged in the soft bottom substrate. Body and head morphologies similar to the PB-morph have been described earlier for small-sized profundal benthivore morphs of Arctic charr (Hesthagen et al. 1995; Alekseyev et al. 2002; Klemetsen et al. 2002; Klemetsen 2010) and from deep-water European whitefish morphs (Kahilainen and Østbye 2006; Siwertsson et al. 2013). The differences in morphology between the LO-morph and the PB-morph are similar as found in a parallel Arctic charr morph pair in Fjellfrøsvatn, Norway, where experimental studies have confirmed heritability of both morphological and trophic behavioral traits (Klemetsen et al. 2002, 2006). Large eye size was a characteristic for both profundal morphs in the present study, but the PB-morph had significantly larger eyes relative to head size compared to the other morphs. Foraging on small prey in low-light environments such as the profundal zone may lead to adaptations toward larger eye size (Huber et al. 1997; Schliewen et al. 2001). Large eyes are also typical for other profundal morphs of Arctic charr (Knudsen et al. 2006; Klemetsen 2010) and European whitefish (Kahilainen and Østbye 2006; Siwertsson et al. 2010) from the same geographical region.

Juvenile Arctic charr often use the profundal habitat in lakes as a refuge from predation (Klemetsen et al. 1989; Sandlund et al. 1992). Typically, they also exhibit morphological adaptations and colorations of an epibenthic feeder with dark dorsal sides, light yellow coloration on lateral sides with darker parr marks, and a blunt snout shape (Skúlason et al. 1989; Klemetsen et al. 2003). The profundal PB-morph seems to retain these juvenile traits into adulthood. Such developmental restrictions are defined as paedomorphism and are well known from many fish taxa (Winterbottom 1990; Hastings 2002), including a few cases of small-sized Arctic charr morphs (Balon 1980; Jonsson et al. 1988; Skúlason et al. 1989; Klemetsen et al. 1997). Paedomorphism is suggested to be an important factor in the local diverging process for these profundal small-sized morphs (Klemetsen et al. 1997). However, the paedomorphic appearance is not necessarily inherited to the next generation. When offspring of the profundal morph in Fjellfrøsvatn were given better foraging conditions, they doubled their growth rate and appeared as typical charr (Klemetsen et al. 2002). Thus, the restricted ecological conditions of the profundal habitat (e.g., reduced nutrients, low prey diversity, and low temperatures) seem to promote paedomorphism (Moore 1994; Klemetsen 2010).

The upper-water omnivore LO-morph was characterized by many traits typical for fish in shallow benthic habitats (Jonsson and Jonsson 2001; Harrod et al. 2010), such as large body depths (anterior, posterior, and caudal peduncle), a robust head (short and deep), and small eyes. However, the silvery coloration, short fins, and relatively small mouth of the LO-morph indicated adaptations to planktivore’s feeding behavior in the pelagic habitat (Webb 1984; Jonsson and Jonsson 2001; Robinson and Parsons 2002). Thus, the morphology of the LO-morph appears to be a combination of the morphological dichotomy of typical pelagic versus benthic fish (Webb 1984; Robinson and Parsons 2002). This has also been documented for the similar LO-morph in Fjellfrøsvatn, where individuals exhibit either typical planktivorous or benthivorous morphologies (Knudsen et al. 2011), closely related to their individual niche use (Knudsen et al. 2014). The LO-morph in Skogsfjordvatn is caught in high density in both the littoral and pelagic habitats, feeding on both zooplankton and benthic prey (Skoglund et al. 2013; Smalås et al. 2013; this study). Such omnivorous feeding behavior is also common for charr in monomorphic populations, especially when there is strong competition for benthic resources in the littoral zone (Jørgensen and Klemetsen 1995; Knudsen et al. 2010; Eloranta et al. 2013). Hence, the zooplanktivore pelagic lifestyle of the LO-morph in Skogsfjordvatn (Skoglund et al. 2013) is probably enforced by strong interspecific resource competition from brown trout and three-spined sticklebacks in the littoral zone.

In Arctic charr, the existence of a single profundal morph living sympatrically with shallow-water morphs has previously been described from various parts of the Northern Hemisphere (Hesthagen et al. 1995; Klemetsen et al. 1997; Alekseyev and Pichugin 1998; Power et al. 2005, 2009; Klemetsen 2010). However, the presence of two morphologically distinct deep-living charr morphs in Skogsfjordvatn represents a novelty in Arctic charr polymorphism, which to our knowledge has not been described previously. The unique evolution of two morphs in the deep-water habitat may be related to two alternative strategies to survive in an environment with constant low temperatures and lean foraging conditions for fish. Low temperature generally leads to slow growth rate in fish, and the two profundal morphs have lower growth rates than the LO-morph in Skogsfjordvatn (Smalås et al. 2013). However, the two morphs have adopted strikingly different strategies in the investment trade-off between somatic growth and reproduction. The PB-morph matures at a small size and a young age (∼8.5 cm/3 years), and when maturation occurs, the somatic growth seems to level off (Smalås et al. 2013). Such limitations in somatic growth following maturation may result in a paedomorphic appearance (Balon 1980; Jonsson et al. 1988). The PP-morph matures at a large size and old age (∼29 cm/9 years) and continues to grow also after maturation (Smalås et al. 2013). This is a common strategy for piscivorous Arctic charr, for which it is important to grow fast to a size where piscivory is possible (Fraser et al. 1998).

The present study confirmed the presence of three sympatric morphs within the Arctic charr population in Skogsfjordvatn. The divergence in body and head morphology between the three morphs seems to correlate functionally to their respective habitat (shallow and deep waters) and trophic niche utilization (i.e., omnivory, benthivory, and piscivory). Correlations between morphology and trophic ecology have been found in several monomorphic and polymorphic populations of *Salvelinus* spp. (e.g., Snorrason et al. 1994; Adams et al. 1998; Knudsen et al. 2007; Woods et al. 2013). Many of the classic polymorphic Arctic charr systems (Sandlund et al. 1992; Adams et al. 1998; Klemetsen 2010) show similar trophic morphologies as the morphs in Skogsfjordvatn, but none of these lakes have a deep-water adapted piscivore morph. Living in contrasting habitats such as in the littoral and the profundal zones results in strong divergent selection due to different environmental factors (Schluter 2001, 2009), and the largest morphological differences were indeed found between morphs residing in different habitats. The two deep-water morphs have evolved a set of morphological similarities, the most obvious being the large head size with relatively large eyes. However, these two morphs also exhibit some large differences, especially in respect to body size and morphological traits related to food acquisition such as head shape and mouth size and position. This evolution of morphological differences within the same habitat (i.e., under similar abiotic conditions) highlights the potential of biotic factors and ecological interactions to promote further divergence between morphs. The diversity of profundal charr in Skogsfjordvatn represents a novelty in the Arctic charr polymorphism as a truly deep-water piscivore morph has to our knowledge not been described elsewhere. Nevertheless, the Arctic charr in Skogsfjordvatn still holds many unsolved mysteries, which calls for further investigations.
